# Multi‐scale effects of habitat loss and the role of trait evolution

**DOI:** 10.1002/ece3.10799

**Published:** 2024-01-04

**Authors:** Rishabh Bagawade, Koen J. van Benthem, Meike J. Wittmann

**Affiliations:** ^1^ Department of Theoretical Biology, Faculty of Biology Bielefeld University Bielefeld Germany; ^2^ Groningen Institute for Evolutionary Life Sciences Faculty of Science and Engineering, University of Groningen Groningen The Netherlands; ^3^ Joint Institute for Individualisation in a Changing Environment (JICE), University of Münster and Bielefeld University Bielefeld Germany

**Keywords:** consumer‐resource systems, eco‐evolutionary dynamics, habitat loss, overexploitation, spatial scales, two‐patch model

## Abstract

Habitat loss (HL) is a major cause of species extinctions. Although the effects of HL beyond the directly impacted area have been previously observed, they have not been modelled explicitly, especially in an eco‐evolutionary context. To start filling this gap, we study a two‐patch deterministic consumer‐resource model, with one of the patches experiencing loss of resources as a special case of HL. Our model allows foraging and mating within a patch as well as between patches. We then introduce heritable variation in consumer traits related to resource utilization and patch use to investigate eco‐evolutionary dynamics and compare results with constant and no trait variation scenarios. Our results show that HL in one patch can indeed reduce consumer densities in the neighbouring patch but can also increase consumer densities in the neighbouring patch when the resources are overexploited. Yet at the landscape scale, the effect of HL on consumer densities is consistently negative. Patch isolation increases consumer density in the patch experiencing HL but has generally negative effects on the neighbouring patch, with context‐dependent results at the landscape scale. With high cross‐patch dependence and coupled foraging and mating preferences, local HL can sometimes even lead to landscape‐level consumer extinction. Eco‐evolutionary dynamics can rescue consumers from such extinction in some cases if their death rates are sufficiently small. More generally, trait evolution had positive or negative effects on equilibrium consumer densities after HL, depending on the evolving trait and the spatial scale considered. In summary, our findings show that HL at a local scale can affect the neighbouring patch and the landscape as a whole, where heritable trait variation can, in some cases, alleviate the impact of HL. We thus suggest joint consideration of multiple spatial scales and trait variation when assessing and predicting the impacts of HL.

## Graphical abstract

We use a two‐patch deterministic consumer‐resource model to study how local habitat loss propagates to another location, and to the landscape scale, in presence and absence of eco‐evolutionary dynamics. We show that local resource loss can drive consumers extinct at the landscape level, although this can be prevented by evolutionary rescue in some cases when the consumer death rate is sufficiently small.

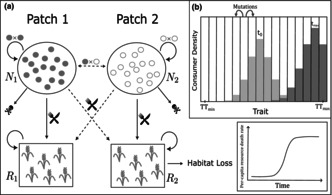

## 1 INTRODUCTION

Habitat loss (HL) is one of the leading causes of biodiversity decline (Fahrig, 2003; McWilliams et al., 2019), and its effects often extend beyond the directly affected habitat. For example, otherwise viable habitat fragments can be affected by the degradation of the surrounding matrix or buffer zone, as shown both empirically (Bierregaard et al., 1992; Friesen et al., 1995) and theoretically (e.g. due to increased mortality in the matrix, Cantrell & Cosner, 1999; Cantrell et al., 1998). Effects may also take place at larger spatial scales. For example, in migratory populations, HL at a single wintering site can affect the population densities in the summer breeding sites due to increased competition in the remaining winter habitat with the displaced individuals (Sutherland & Dolman, 1994). Local HL can reduce habitat connectivity, culminating in extinctions at both local and regional scales (Horváth et al., 2019). Furthermore, local HL can affect the stability of the entire community, for example, by constraining the mobility of the remaining community in a smaller region, leading to increased encounter rates and thereby increasing the interaction strengths and destabilizing the system (McWilliams et al., 2019). This tendency of HL effects to go beyond the local spatial scale necessitates the study of HL effects over multiple spatial scales.

Besides affecting ecological properties such as interaction strengths and population stability, HL may also affect evolutionary dynamics by altering phenotypic trait variation and the underlying genetic diversity, which in turn may affect ecological processes. Genetic diversity, for example, can be reduced by HL through a reduction in reproductive output and an increase in inbreeding (Lowe et al., 2005). Furthermore, HL can influence the evolution of species either directly by differential loss of specific types of habitats and associated changes in selection pressures or indirectly by reducing population sizes and thereby also reducing genetic diversity (McClure et al., 2008). A reduction in genetic diversity can even trigger an extinction vortex if the populations become too small (Gilpin & Soulé, 1986; Nabutanyi & Wittmann, 2021, 2022). HL can also influence evolution, for example, of dispersal distance, such that removal of entire patches would select for reduced dispersal, but degradation (reducing carrying capacity) of patches would select for longer dispersal in a multi‐patch landscape (North et al., 2011).

Conversely, trait variation can play a role in mitigating the population‐dynamic consequences of HL and environmental change. For example, genetic and phenotypic diversity tend to reduce the vulnerability of populations to environmental change (reviewed in Forsman & Wennersten, 2016), and diversity in individual behavioural traits (risk taking vs. avoiding) can promote species coexistence in communities experiencing HL (Rohwäder & Jeltsch, 2022). Furthermore, when the trait variation is heritable, it can also help mitigate the effects of environmental change through ‘evolutionary rescue’ (Bell & Gonzalez, 2011; Boeye et al., 2013; Gonzalez et al., 2013), or conversely aggravate the negative effects through ‘evolutionary trapping’ (Ferriere & Legendre, 2013). These reciprocal effects can lead to eco‐evolutionary feedbacks between the population dynamic consequences of HL and trait variation, which are increasingly being recognized (Faillace et al., 2021; Gawecka et al., 2022; Legrand et al., 2017).

Apart from the interplay between the ecological effects of HL and trait variation, multi‐scale spatial processes can also interact with trait variation and evolution, thereby influencing population dynamics. For example, in a two‐patch model, multi‐scale density dependence, where density in one patch influences the fitness of individuals in the adjacent patch and vice versa, can lead to adaptation to emerging low‐ and high‐density patches (van Benthem & Wittmann, 2020). Moderate immigration from a nearby source patch can provide the necessary genetic material for local adaptation and eventually lead to evolutionary rescue in a sink habitat (Gomulkiewicz et al., 1999). Spatial processes such as range expansion can lead to bursts of evolutionary change under genetic drift or even directional evolution under spatially structured selection gradients (Polly, 2019). Furthermore, spatially structured intraspecific trait variation can promote species coexistence when species' responses to habitat conditions are different (Banitz, 2019).

In summary, HL affects populations over multiple spatial scales, and these effects can be altered by trait variation. The interplay between HL, multi‐scale interactions, and trait variation can lead to eco‐evolutionary dynamics. In this study, we use the term eco‐evolutionary dynamics in the broader sense where there is at least a one‐way interaction between ecological and evolutionary dynamics (as defined in Hendry, 2017). Currently, such eco‐evolutionary processes for interacting populations exposed to HL are not well understood. Given that mathematical models can act as a proof of concept to address such complex phenomena (Servedio et al., 2014), we take a step in that direction by modelling a deterministic two‐patch consumer‐resource system with two spatial scales: local (patch‐level) and landscape (both patches combined). In our model, consumers can mate and forage within and between patches, with one of the patches experiencing HL. Such a system is relevant to regionally confined resources with more freely moving consumer populations and where resources are the primary food source, such that resource loss is an important form of HL. Example systems could be coral (resource) and coral‐associated fish (consumer), where the fish can move between the coral reefs to forage or mate (Bonin et al., 2011), and krill (resource) and Antarctic fur seals (consumer), where the seals can roam into the sea for food and come back to the islands to birth pups (Boyd, 2002). We explore how HL effects propagate from local to landscape scale, how effects of HL change due to the presence of a nearby unharmed patch, and whether heritable trait‐variation in consumer traits helps mitigate these effects through eco‐evolutionary dynamics.

## 2 METHODS

**FIGURE 1:**
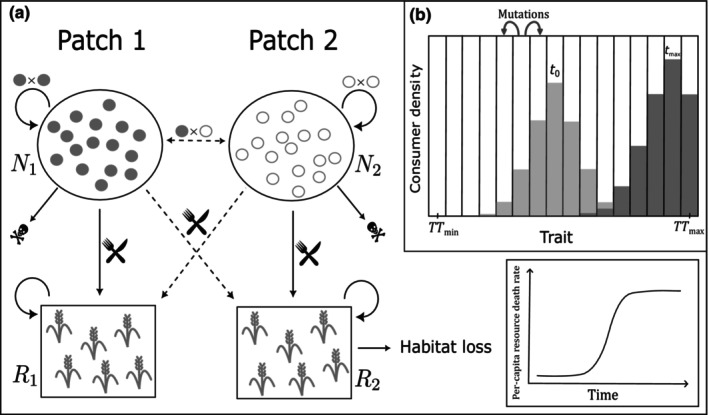
A schematic overview of the model. (a) Schematics of the two patch systems with consumers and resources. Dashed arrows indicate cross‐patch interactions (foraging or mating). In the presence of habitat loss, resources in patch 2 are depleted through a logistically increasing resource death or removal rate. (b) In the presence of trait variation, populations are divided into bins and their growth rate depends on the trait value assigned to that bin. Equidistant trait values are assigned to each bin, such that the first bin gets ${\mathrm{TT}}_{\min}$ and the last bin gets ${\mathrm{TT}}_{\max}$ as the trait value. Mutations are modelled such that a fraction μ (mutation rate) of the offspring density from each bin is equally redistributed in its adjacent bins. Example trait distributions are shown for initial (at $t_{0}$, light grey) and final (at $t_{\max}$, dark grey) time points. The trait distribution is kept constant in the case of constant variation, and it evolves in the case of heritable variation. Note that the no‐variation scenario is equivalent to there being only one bin with the trait value equal to the initial mean trait value of the other two variation scenarios.

We use a system of coupled ordinary differential equations (ODEs) to describe a two‐patch model where each patch contains a consumer population and a resource population (Figure 1a). We define $R_{1}$ and $R_{2}$ as the resource densities in patches 1 and 2, respectively. $N_{1}$ and $N_{2}$ are defined as the densities of consumers born in patch 1 and patch 2, respectively. Temporary movement between patches by an individual within its lifetime has no effect on $N_{1}$ and $N_{2}$. Such movement arises during cross‐patch foraging, where individuals look for resources in the other patch, and cross‐patch mating, where individuals mate with partners from the other patch. However, foraging and mating impacts the growth rate of consumers, which in turn can change $N_{1}$ and $N_{2}$. Resources, by contrast, do not move between patches. We model habitat loss (HL) as the death or removal of the resources in patch 2 because of external anthropogenic factors such as harvesting, contamination, or degradation of resources. Loss of resources has been considered a form of HL in previous studies, for example in the experimental manipulation studies of coral and coral‐associated fish systems, where the removal of coral was considered as HL for the fish (Bonin et al., 2011). Consumers are the species of interest whose fate under HL is investigated in our study. We further assume that HL does not cut off the access of consumers to that patch; that is, consumer breeding sites can still exist in that patch. Another important assumption is that cross‐foraging and mating preferences do not change after HL unless allowed to evolve under the eco‐evolutionary model. Below, we define a purely ecological model and then extend this model by including eco‐evolutionary dynamics.

### 2.1 Ecological model

The resources are replenished according to a logistic growth model:

(1) $$G_{i}=r_{0}R_{i}\left(1-\frac{R_{i}}{k}\right),$$

where $G_{i}$ is the population growth rate for the resource in patch $i\in \left\{1,2\right\}$, $r_{0}$ is the maximum per‐capita growth rate, and k is the carrying capacity. All parameters with their default values are noted in Table 1.

**TABLE 1:** Parameters, their description, intervals of possible values and default values.

Parameter	Description	Interval	Default value
$a_{0}$	Per‐capita death rate	$\left[0,\infty \right]$	0.1
$b_{0}$	Maximum per‐capita resource consumption rate	$\left[0,\infty \right)$	30
$b_{1}$	Half‐saturation constant for resource consumption	$\left[0,\infty \right)$	500
p	Within‐patch foraging preference	$\left[0,1\right]$	0.75
β	Within‐patch mating preference	$\left[0,1\right]$	0.75
θ	Allee effect parameter	$\left[0,\infty \right)$	2
ϵ	Per‐capita resource conversion efficiency	$\left[0,\infty \right)$	0.2
$\epsilon_{c}$	Cross‐patch foraging efficiency relative to within‐patch	$\left[0,1\right]$	0.9
$r_{0}$	Maximum growth rate of resources	$\left[0,\infty \right)$	0.5
k	Carrying capacity of resources	$\left[0,\infty \right)$	50
D	Maximum rate of resource degradation or removal	$\left[0,\infty \right)$	0.5
s	Steepness of logistic resource removal	$\left[0,\infty \right)$	0.003
$t_{1/2}$	Time when the resource removal rate is D/2	$\left[0,\infty \right)$	3500
$N_{0,1},N_{0,2}$	Initial consumer densities for patch 1, patch 2	$\left[0,\infty \right)$	4, 3
$R_{0,1},R_{0,2}$	Initial resource densities for patch 1, patch 2	$\left[0,\infty \right)$	10, 10
μ	Mutation rate	$\left[0,1\right]$	0.3
B	Number of bins when trait variation is present	ℕ	21

Assuming a Michaelis–Menten like formulation of a type II functional response, the per‐capita resource consumption rate by consumers in patch i of the resource in patch j, $C_{\mathrm{ij}}$, is
(2)

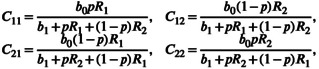

where $b_{0}$ is the maximum per‐capita resource consumption rate, $b_{1}$ is the half‐saturation constant, p is the within‐patch foraging preference, and consequently $\left(1-p\right)$ is the cross‐patch foraging preference. These preferences are proportions and are thus between 0 and 1. Note that ${\mathrm{pR}}_{1}+\left(1-p\right)R_{2}$ corresponds to the average resource density weighted by the amount of within‐patch and cross‐patch foraging for consumers in patch 1 (analogously, ${\mathrm{pR}}_{2}+\left(1-p\right)R_{1}$ for patch 2 consumers).

As mentioned earlier, we model HL via resource loss in patch 2 at the rate

(3) $$H_{R_{2}}=\frac{D}{1+e^{-s\left(t-t_{1⁄2}\right)}}R_{2},$$

where D is the maximum rate of resource removal, $t_{1/2}$ is the time at which the resource removal rate is D/2, and s controls the steepness (i.e., how sudden the resource removal rate changes around $t_{1/2}$). Thus, resource removal starts slowly when t=0, then becomes increasingly fast, before finally approaching a maximum (Figure 1a). See SI Section S1 for further discussion about the shape of HL function ($H_{R_{2}}$). For the scenarios without HL, D=0 and thus $H_{R_{2}}=0$. Taking everything together, the ecological dynamics of resources in the two patches are given by:

(4) $$\begin{array}{c} \frac{{\mathrm{dR}}_{1}}{\mathrm{dt}}=G_{1}-C_{11}N_{1}-C_{21}N_{2},\\ \frac{{\mathrm{dR}}_{2}}{\mathrm{dt}}=G_{2}-C_{22}N_{2}-C_{12}N_{1}-H_{R_{2}}. \end{array}$$

Consumer population growth rates in patches 1 and 2 are modelled as:

(5) $$\begin{array}{l} F_{1}=\epsilon \cdot A_{1}\cdot \left(C_{11}+\epsilon_{c}\cdot C_{12}\right)\cdot \frac{N_{1}}{2},\\ F_{2}=\epsilon \cdot A_{2}\cdot \left(C_{22}+\epsilon_{c}\cdot C_{21}\right)\cdot \frac{N_{2}}{2}, \end{array}$$

where ϵ is the efficiency of resource conversion, $\epsilon_{c}\in \left[0,1\right]$ is the cross‐patch foraging efficiency relative to the within‐patch foraging efficiency (assuming individuals spend more energy per unit time foraging in the other patch compared to foraging in their own patch), $A_{i}$ is a mate finding Allee effect term (see below) in patch i, $C_{\mathrm{ij}}$ are the per‐capita consumption rates as defined in Equation (2). Assuming a constant 1:1 sex ratio, the population densities ($N_{i}$) are divided by a factor of 2 to model that only half of the population (females) gives birth.

When HL leads to reduced population sizes, populations may experience an Allee effect (Swift & Hannon, 2010), that is, a decrease in survival and/or reproduction with decreasing population size in small populations, for example, because it becomes more difficult to find mating partners (Fauvergue, 2013). Since we have a continuous model where population densities can get in principle arbitrarily small, the Allee effect also prevents unrealistic increases from very low densities and allows for true extinctions. We model a mate‐finding Allee effect, where the probability of finding at least one male mating partner if there are M available males as $1-e^{-\mathrm{\theta M}}$ (Dennis, 1989; Fauvergue, 2013). Here, θ is inversely proportional to the strength of the Allee effect. To determine the value of M, we have to account for cross‐patch mating. We define the within‐patch mating preference as $\beta \in \left[0,1\right]$, and consequently 1−β equals the cross‐patch mating preference. The number of males that a female sees is determined as the average of males in her own patch and in the other patch, weighted by β and 1−β, respectively. The probability of at least one successful mating event for a female in patch i, $A_{i}$, thus becomes:
(6)

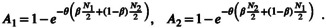




We define $q_{\mathrm{ij}}$ as the fraction of newborns produced by females in patch i via mating with males in patch j as:

(7) $$\begin{array}{cc} q_{11}=\beta N_{1}/\left(\beta N_{1}+\left(1-\beta \right)N_{2}\right), & q_{12}=\left(1-\beta \right)N_{2}/\left(\beta N_{1}+\left(1-\beta \right)N_{2}\right),\\ q_{22}=\beta N_{2}/\left(\beta N_{2}+\left(1-\beta \right)N_{1}\right), & q_{21}=\left(1-\beta \right)N_{1}/\left(\beta N_{2}+\left(1-\beta \right)N_{1}\right). \end{array}$$

Females that mate within patch are assumed to produce their offspring in their own patch, whereas females that engage in cross‐patch mating are assumed to be equally likely to produce their offspring in either of the patches. This means that for patch 1, offspring coming from within‐patch mating $q_{11}F_{1}$ stays in patch 1, whereas half of the offspring from cross‐patch mating (i.e. $\frac{q_{12}}{2}F_{1}$) stay in patch 1, and the other half go to patch 2. The dynamics for patch 2 happen analogously. Lastly, consumers die with a constant per‐capita death rate $a_{0}$. Therefore, the consumer ecological dynamics are:

(8) $$\begin{array}{l} \frac{{\mathrm{dN}}_{1}}{\mathrm{dt}}=q_{11}F_{1}+\frac{q_{12}}{2}F_{1}+\frac{q_{21}}{2}F_{2}-a_{0}N_{1},\\ \frac{{\mathrm{dN}}_{2}}{\mathrm{dt}}=q_{22}F_{2}+\frac{q_{21}}{2}F_{2}+\frac{q_{12}}{2}F_{1}-a_{0}N_{2}. \end{array}$$

The resource dynamics in Equation (4) and consumer dynamics in Equation (8) together form the full ecological model.

### 2.2 Eco‐evolutionary model

We now add heritable trait variation and mutations to the consumer population such that the consumer parameters are considered as traits (similar to van Benthem & Wittmann, 2020). We investigated intraspecific variation in per‐capita resource conversion efficiency ϵ, maximum per‐capita resource consumption rate $b_{0}$, or half‐saturation constant for resource consumption $b_{1}$, with only one parameter varying at a time. Moreover, we looked at a scenario with two coupled traits: within‐patch foraging preference p and within‐patch mating preference β where both of them vary together such that p=β. This choice makes biological sense, because spending more time foraging in one patch might also lead to a higher chance of finding a mate in the same patch. To better understand how ecological and evolutionary consequences of trait variation affect the population dynamics, we also implement runs with constant trait distribution. Despite lacking evolution, this scenario may still deviate from the no variation case through non‐linear averaging (Bjørnstad & Hansen, 1994; Ruel & Ayres, 1999).

We assume traits to be determined by an infinite number of loci with alleles of small effect, which would give rise to a continuous trait distribution. We approximate this trait distribution by discretizing it into B bins with a population density of $n_{i,b}$ and trait value $z_{b}$ for the bin b in patch i (Figure 1b). The total consumer density in patch i is thus $N_{i}=\sum_{b=1}^{b=B}n_{i,b}$. Population dynamics for each bin are determined by the population densities in the patches, the resource densities, and the bin‐specific trait value $z_{b}$. The first and last bin correspond to the minimum and maximum trait values and are thus defined as $z_{1}={\mathrm{TT}}_{\min}$ and $z_{B}={\mathrm{TT}}_{\max}$, with equally spaced trait values for the intermediate bins. The initial trait distribution is obtained by truncating and re‐normalizing a normal distribution with mean ${\mathrm{TT}}_{\text{mean}}$ and standard deviation ${\mathrm{TT}}_{\mathrm{SD}}$. That is, to ensure that the initial distribution stays within the defined interval, we set those bins that are beyond ${\mathrm{TT}}_{\min}$ or ${\mathrm{TT}}_{\max}$ to zero, and, to maintain the mean and keep the distribution symmetric, we also set their counterparts on the other side of ${\mathrm{TT}}_{\text{mean}}$ to zero. In the eco‐evolutionary model, the trait distribution evolves over time through the changing number of individuals in the different bins. In the scenarios where p and β are coupled (p=β), each bin now represents two traits of identical value. The default values of ${\mathrm{TT}}_{\text{mean}}$, ${\mathrm{TT}}_{\mathrm{SD}}$, ${\mathrm{TT}}_{\min}$, and ${\mathrm{TT}}_{\max}$ for the evolving parameters are listed in Table 2. In general, ${\mathrm{TT}}_{\text{mean}}$ is equal to the default parameter values from Table 1, and ${\mathrm{TT}}_{\mathrm{SD}}$ is chosen to be half of the difference between ${\mathrm{TT}}_{\text{mean}}$ and the closest end point of the trait range (but see Figure S6 to see the impact of ${\mathrm{TT}}_{\mathrm{SD}}$ on equilibrium consumer densities).

**TABLE 2:** Default values of initial mean trait value ${\mathrm{TT}}_{\text{mean}}$, initial trait standard deviation ${\mathrm{TT}}_{\mathrm{SD}}$, and minimum ${\mathrm{TT}}_{\min}$ and maximum ${\mathrm{TT}}_{\max}$ possible trait value for traits in heritable and constant variation scenarios.

Evolving trait	${\mathrm{TT}}_{\text{mean}}$	${\mathrm{TT}}_{\mathrm{SD}}$	${\mathrm{TT}}_{\min}$	${\mathrm{TT}}_{\max}$
Per‐capita resource conversion efficiency, ϵ	0.2	0.1	0	0.4
Maximum per‐capita resource consumption rate, $b_{0}$	30	7.5	15	45
Half‐saturation constant for resource consumption, $b_{1}$	500	200	100	900
Coupled foraging and mating preference, p=β	0.65	0.17	0	1

Fecundity is determined by the female's trait value. When a female mates, she randomly picks a mating partner in proportion to bin densities, that is, there is no sexual selection and males can mate with an arbitrary number of females. Offspring trait values are determined by the average trait value of the two parents. If the mid‐parent trait value is on the boundary between two adjacent bins, the offspring density is evenly split between these two bins. The offspring trait distribution of any given female therefore depends on the paternal trait distributions in both patches, and on the chances that she mates with males in each of the two patches. In the scenarios with heritable β, the population can evolve for females to have a preference for mating with males in either the own patch or in the other patch. Putting everything together (see SI Section S2 for the technical details), we then obtain $f_{i,b}$, the rate at which offspring are added to bin b in patch i due to reproduction in the whole population. Then, in the absence of mutations, our consumer population would develop as follows:

(9) $$\begin{array}{cc} \frac{{\mathrm{dn}}_{i,b}}{\mathrm{dt}}=f_{i,b}-a_{0}n_{i,b} & \text{for}\;i\in 1,2. \end{array}$$

The trait distribution of the offspring is then further affected by mutations. These are modelled deterministically such that a fraction μ (the mutation rate) of the offspring density in bin b mutates away from the bin and is evenly distributed between bins b+1 and b−1. For the first and the last bin, mutation occurs only in one direction with half the rate (μ/2). Thus, the population dynamics for the bins in patch i are

(10) $$\frac{dn_{i,b}}{dt}=\left\{\begin{array}{ll} f_{i,b}-\mu f_{i,b}+\frac{\mu }{2}f_{i,b-1}+\frac{\mu }{2}f_{i,b+1}-a_{0}n_{i,b}, & \text{if}\;1<b<B\\ f_{i,1}-\frac{\mu }{2}f_{i,1}+\frac{\mu }{2}f_{i,2}-a_{0}n_{i,1}, & \text{if}\;b=1\\ f_{i,B}-\frac{\mu }{2}f_{i,B}+\frac{\mu }{2}f_{i,B-1}-a_{0}n_{i,B}, & \text{if}\;b=B \end{array}\right.$$

where $i\in \left\{1,2\right\}$.

The resource dynamics for the two patches, in presence of trait variation in consumers, are given by

(11) $$\begin{array}{c} \frac{{\mathrm{dR}}_{1}}{\mathrm{dt}}=G_{1}-\sum_{b=1}^{b=B}\left(C_{11,b}n_{1,b}+C_{21,b}n_{2,b}\right),\\ \frac{{\mathrm{dR}}_{2}}{\mathrm{dt}}=G_{2}-\sum_{b=1}^{b=B}\left(C_{22,b}n_{2,b}+C_{12,b}n_{1,b}\right)-H_{R_{2}}, \end{array}$$

where the consumption terms $C_{\mathrm{ij},b}$ depend on the bin b whenever consumer trait values affect the consumption rates.

As a baseline scenario for the eco‐evolutionary case, we also model a constant variation case where the densities in the trait bins are redistributed after every time step such that the trait distribution over time always remains identical to the initial distribution of the eco‐evolutionary model. These models thus disregard both mutations and inheritance. We further set the trait value in the ecological (no variation) scenario to be identical to the mean trait from the initial distribution in the other two scenarios.

The system of equations is solved numerically using the deSolve (Soetaert et al., 2010) package in R (R Core Team, 2022). The numerical solver is run until time 8000 for all the results, unless stated otherwise. We made sure this was long enough for the population densities to stabilize, as confirmed by visual inspection of all underlying time series. Since $a_{0}=0.1$ (in the default settings) is defined as the per‐capita consumer death rate per unit time, $1/a_{0}=10$ roughly correspond to the life‐span of the consumers such that the simulation time corresponds roughly to 800 generations.

We focus on the parameter region where the populations reached stable (non fluctuating) densities by the end of the runs (but see Figure S4 for an example with fluctuating dynamics). Density at the last time point is thus considered as the equilibrium density. We use unequal initial consumer densities for the two patches because the case where they are exactly equal could act as a special case where slight perturbations can change the outcome (see Figure S5). We are also aware that alternative stable states may exist for our system, which is why we compare scenarios with identical initial conditions to ensure comparability between results (see Figure S5). We do not calculate all the fixed points and stability of the full ecological model. However, we do provide analytical treatment for a simplified approximation of the full ecological model in SI Section S3.

### 2.3 Scenarios explored

The scenarios explored in this study are based on presence or absence of three aspects: habitat loss (HL), trait variation, and patch isolation. First, we compare HL and no HL scenarios. Second, we compare no trait variation (ecological model), and heritable variation (eco‐evolutionary model) along with constant variation as the baseline for the eco‐evolutionary model. Lastly, we compare the effects of HL when the patches are connected (this is the default scenario) to when they are isolated. Patch isolation is modelled by assuming that the two patches have no access to each other (because of distance or physical barriers) i.e. we put $N_{2},R_{2}=0$ in the equations of patch 1 dynamics and $N_{1},R_{1}=0$ in the equations of patch 2 dynamics. Here, we assume that the consumers have an inherent preference to go out of the patch (with foraging preference 1−p) but cannot access the resources nor potential mating partners in the other patch.

Using the models and scenarios described above, we investigate the effects of local HL over multiple spatial scales and how eco‐evolutionary dynamics influence these effects. We specifically address the following questions: (1) How does HL in one of the patches affect the consumer densities in that patch, the neighbouring patch, and the landscape as a whole? (2) How does the presence of a neighbouring patch affect the dynamics in the patch experiencing HL? (3) Can eco‐evolutionary dynamics mitigate the multi‐scale effects of HL?

## 3 RESULTS

**FIGURE 2:**
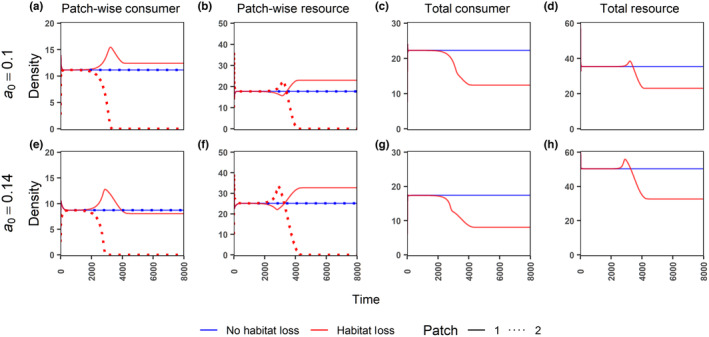
Time‐series plots under the ecological model (no trait variation) for patch‐wise consumer (panels a, e), patch‐wise resource (panels b, f), total (landscape scale) consumer ($N_{1}+N_{2}$, panels c, g) and total resource ($R_{1}+R_{2}$, panels d, h) densities in the presence or absence of habitat loss. Note that for panels c, d, g, h solid lines imply total densities (i.e., there is no dotted line). All parameters are at their default values from Table 1, except for the per‐capita consumer death rate $a_{0}$ whose values are mentioned for each row.

We start with the ecological model, for which we compare the dynamics with and without habitat loss (HL) (Figure 2). After sufficient time, the consumer and resource densities equilibrate. When HL is absent (blue lines), densities in patches 1 and 2 are equal at equilibrium. In the presence of HL, the resources in patch 2 start to decline and disappear completely around time 4000 (Figure 2b,f, red dotted lines), with the consumers in patch 2 already going extinct well before time 4000 (Figure 2a,e, red dotted lines). As a consequence, between time 2000 and 4000, there is a transient increase in resource density in patch 2 and consequently in consumer density in patch 1 because there is no more competition from patch 2 consumers.

Resource degradation in patch 2 may lead to higher patch 1 equilibrium consumer density when the consumer death rate $a_{0}$ is low (Figure 2a, observe red solid line above the blue line at equilibrium), but not when $a_{0}$ is high (Figure 2e). This is counterintuitive because when the resources in patch 2 are lost, patch 1 consumers can no longer cross‐forage, and thereby the effective per‐capita resource consumption must reduce, irrespective of $a_{0}$ (SI Section S4). However, when resources are overexploited in a consumer‐resource system, a reduction in per‐capita consumption can increase the consumer density (see Box 1). Thus, we infer that the system is in the overexploitation regime at low $a_{0}$ (0.1), where the HL‐induced reduction in effective consumption rate leads to an increase in consumer density (Figure 2a). At higher $a_{0}$, the system leaves the overexploitation regime such that HL leads to lower consumer densities in patch 1 (Figure 2e). Importantly, irrespective of the observations for individual patches, the total consumer (and resource) density at the landscape levels is always lower after HL (Figure 2c,d,g,h).

BOX 1 **Overexploitation**

Under overexploitation, a reduction in resource consumption by consumers can lead to an increase in equilibrium consumer density. This counter‐intuitive outcome occurs when the consumers are initially highly efficient, that is, resources are overexploited, such that the resource growth rate is below its optimum. When the resource consumption rate now declines, the consumption pressure on the resources is eased and an increase in resource growth is observed. Such overexploitation is observed in consumer‐resource systems where the resources have a logistic growth rate (or other functions where resource growth is maximal at intermediate resource density) and the consumers are highly efficient (Abrams, 2002, 2009, 2019).

**FIGURE B1:**
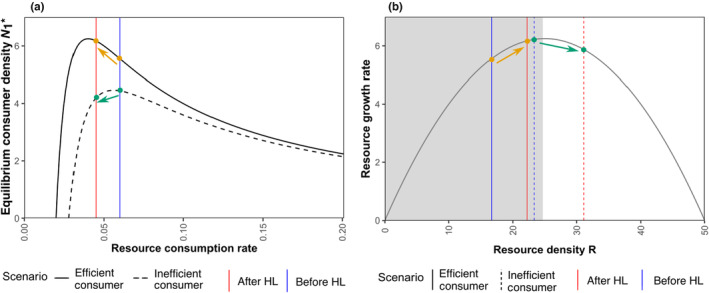
Schematic figure explaining overexploitation where the equilibrium consumer density in the remaining patch ($N_{1}^{*}$) increases after HL (solid lines in (a) and orange arrows) for efficient consumers (here, low consumer death rate) and the equilibrium consumer density decreases after HL (dashed lines in (a) and green arrows) for inefficient consumers (here, high consumer death rate). (a) Shows the equilibrium consumer density of patch 1 as a function of resource consumption rate, where the vertical lines denote the resource consumption rate before HL (blue line) and after HL (red line). Here, the region on the right of the peak of the curves represents the overexploitation regime. One can observe that resource consumption reduces after HL, leading to an increase in consumer density under overexploitation (orange arrow) and an effective decrease (green arrow) in the density as the system leaves the overexploitation regime. (b) Shows the resource growth rate as a function of resource density (logistic growth model), where the shaded region on the left of the peak is where the resources are overexploited (overexploitation regime). The vertical lines denote the equilibrium resource density for the corresponding consumer type and HL scenario from panel (a). One can observe that the orange arrow stays in the overexploitation regime such that resource growth rate increases after HL, whereas the green arrow starts under overexploitation but leaves the regime after HL such that there is an effective decrease in resource growth rate.

In our model, when HL occurs under the default scenario, patch 2 consumers and resources are lost completely. This stops the cross‐foraging by patch 2 consumers on patch 1 resources, and at the same time, patch 1 consumers still attempt cross‐foraging with preference (1−p), while within patch foraging preference remains p (where 0≤p≤1). In other words, HL leads to a reduction in overall consumption of patch 1 resources, and hence we see a decline in resource consumption after HL. Therefore, when the system is in the overexploitation regime, HL leads to an increase in patch 1 consumer density, whereas HL leads to a decrease in patch 1 consumer density when the resources are not overexploited anymore (see SI Section S4 for detailed explanation). Figure B1 schematically explains this phenomenon, where the orange arrows represent the overexploitation scenario where consumer density in the remaining patch increases after HL. The green arrows represent a scenario where the resources are overexploited before HL, but the system leaves the overexploitation regime after HL such that the consumer density in the remaining patch decreases. Panel (a) shows how equilibrium consumer density changes for the two scenarios with a decrease in resource consumption, whereas panel (b) shows corresponding changes in resource growth rate for the two scenarios.

**FIGURE 3:**
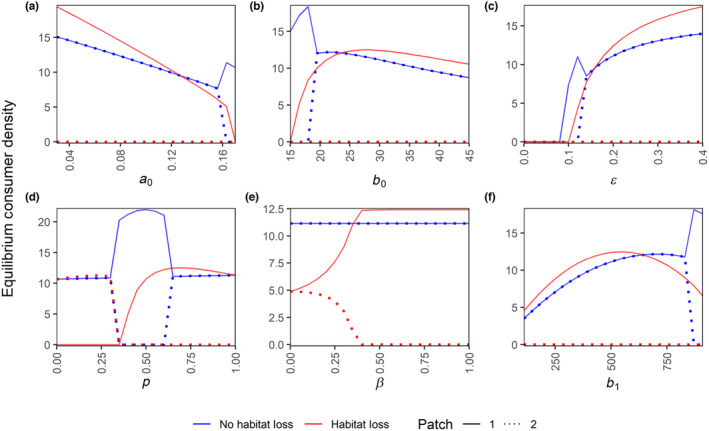
Patch‐wise equilibrium consumer densities for the ecological model (no trait variation) over certain parameter ranges of: (a) per‐capita death rate $a_{0}$, (b) maximum consumption rate $b_{0}$, (c) resource conversion efficiency ϵ, (d) within‐patch foraging preference p, (e) within‐patch mating preference β, and (f) half‐saturation constant for resource consumption $b_{1}$. Note that the equilibrium densities are calculated for 21 equidistant points in each parameter range, but they are depicted by lines for better clarity. All other parameters are at their default values from Table 1.

Whether HL in patch 2 increases or decreases the equilibrium density in patch 1 depends on the values of several parameters. This is shown in Figure 3 by the crossing of the lines for patch 1 equilibrium density for the scenarios with HL (red solid lines) and those without (blue solid lines) for the parameters $a_{0}$, $b_{0}$, ϵ, p, β, and $b_{1}$ (Figure 3). For $a_{0}$, $b_{0}$, $b_{1}$, and ϵ this effect (crossing of red and blue solid lines) can be explained using overexploitation. When the consumers are highly efficient (low $a_{0}$ or $b_{1}$, or high $b_{0}$ or ϵ) the system is in the overexploitation regime, and thus consumer density in patch 1 increases after HL (see Box 1). In contrast, when the consumers are inefficient (high $a_{0}$ or $b_{1}$, or low $b_{0}$ or ϵ), the system is out of the overexploitation regime, and hence the consumer density in patch 1 decreases after HL. Close to the crossing point of red and blue solid lines, HL can move the system out of an overexploitation regime and the effect of consumer density depends on how far HL moves the system out of the regime (see Box 1, and SI Section S4). For β this happens because, at lower values of β, consumers in both patches survive after HL. However, patch 1 consumers have to suffer in this case since consumers in both patches are now dependent on patch 1 resources. For p this happens because at the values between approximately 0.3 and 0.6, patch 1 consumers outcompete patch 2 consumers in the scenario without HL. For p greater than this range, both patches coexist again, which reduces consumer density in patch 1 as consumers are now present in both patches.

In certain parameter regions, we observe outcompetition by the consumers that have higher initial density (here patch 1 consumers, but see Figure S5 to observe how it depends on the initial consumer density). This occurs particularly in the regions where the consumers are less efficient i.e. higher values of $a_{0}$ and $b_{1}$ (Figure 3a,f), and lower values of $b_{0}$ and ϵ (Figure 3b,c). Similar outcompetition also occurs at p values between approximately 0.3 and 0.6 (Figure 3d). At more extreme values of p (i.e. below 0.3 or above 0.6), the larger initial population size in patch 1 no longer leads to outcompetition. Instead, at these extreme values of p, the consumers that forage least in patch 2 and most in patch 1 survive after HL; this will be patch 1 consumers when p is high and patch 2 consumers when it is low. Apart from outcompeting each other, the consumers in the two patches can also help each other survive by subsidizing the other patch because half of their offspring from cross‐patch mating is put in the other patch. This happens at lower values of within‐patch mating preference β, where both patches survive after HL (Figure 3e). We also assess the impact of Allee effect under HL over the parameter ranges in Figure 3 and find that even a slight Allee effect (here, the default θ=2) shows earlier extinctions when the consumer are less efficient (i.e. high death rate ($a_{0}$), low maximum resource consumption rate ($b_{0}$), and low resource conversion efficiency (ϵ)) (Figure S8).

**FIGURE 4:**
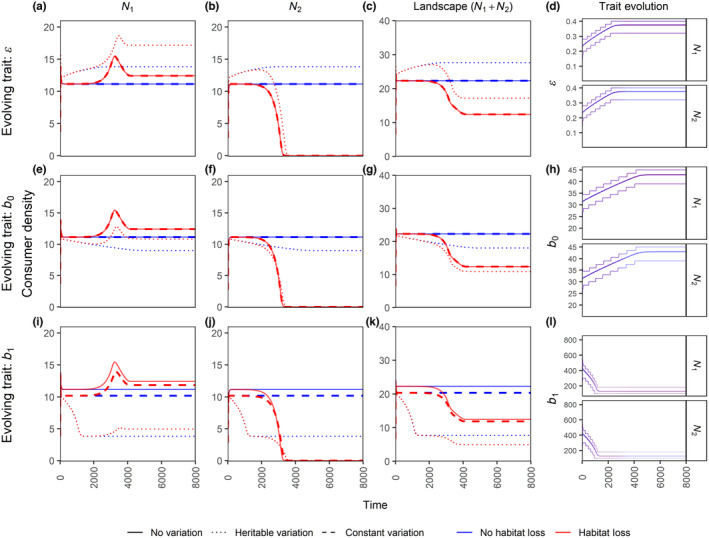
Time‐series plots for patch 1 (panels a, e, i), patch 2 (panels b, f, j) and landscape level (panels c, g, k) consumer densities for the three variation scenarios in the presence and absence of habitat loss. The evolving trait is resource conversion efficiency ϵ for the top row, maximum resource consumption rate $b_{0}$ for the middle row, and half‐saturation constant for resource consumption $b_{1}$ for the bottom row. The last column (panels d, h, l) shows the trait evolution where the central line denotes the mean trait value and the thinner outer lines denote the range which contains 90% of the density. Line transparency in the trait evolution plots denote consumer density. Note that the red and blue lines are overlapping in the trait evolution plots. All other parameter values are at their default values from Tables 1 and 2.

Including constant trait variation in the ecological model does not necessarily lead to qualitative changes in population dynamics (Figure 4). For example, when variation is present in resource conversion efficiency ϵ or maximum resource consumption rate $b_{0}$, the constant variation (dashed lines) and no variation (solid lines) scenarios are indistinguishable (Figure 4 top and middle row). Both ϵ and $b_{0}$ affect the growth rate linearly, and the mean trait value for the two scenarios is always identical. Therefore, the effect of constant variation is not expected to be different from no variation (Ruel & Ayres, 1999). However, the dynamics of the no variation and constant variation cases differ (even with identical mean trait values) if there is non‐linear dependence between growth rate and the trait, for example when trait variation is present in $b_{1}$ (Figure 4 bottom row; also see Figure S6 for the impact of the amount of trait variation ${\mathrm{TT}}_{\mathrm{SD}}$). This can happen through non‐linear averaging (Bjørnstad & Hansen, 1994; Ruel & Ayres, 1999).

When the trait variation is heritable (eco‐evolutionary scenario), even variation in traits with a linear effect on the growth rate can lead to either higher or lower equilibrium densities than the other two variation cases, depending upon which trait is allowed to evolve. The trait evolves to either higher or lower values, thereby maximizing individual consumer growth. For example, based on Equations (2) and (5), one can predict that ϵ and $b_{0}$ would evolve to higher values, whereas $b_{1}$ would evolve to lower values if consumers maximize their individual growth. Therefore, the selection pressure on these traits is independent of whether there is HL, and hence they have the same evolutionary trajectory in presence as in the absence of HL. Furthermore, when $b_{0}$ and $b_{1}$ evolve, the equilibrium density is lower in the eco‐evolutionary model (Figure 4 middle and bottom row, dotted vs. solid line), while it is higher when ϵ evolves (Figure 4, top row). The different behaviour arises because $b_{0}$ and $b_{1}$ influence the per‐capita consumption rate, whereas ϵ only influences consumer efficiency. This shows the impact of evolutionary dynamics (change in trait value) on ecological dynamics (impact on population density) such that evolution of $b_{0}$ or $b_{1}$ leads to more overexploitation and thereby decreases consumer density, whereas an evolution of ϵ just benefits consumer growth and thereby increases consumer density (also see Figure 3b,c,f to observe how equilibrium consumer density changes with these three parameters).

**FIGURE 5:**
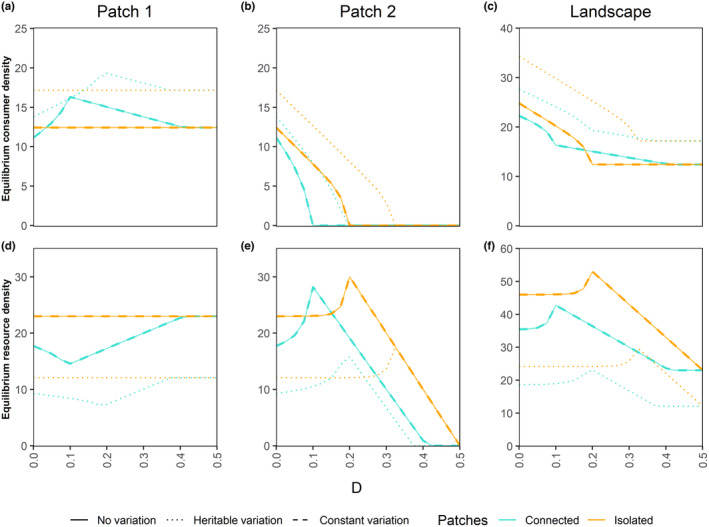
Patch 1 (panels a, d), patch 2 (panels b, e) and landscape scale (panels c, f) equilibrium consumer (row 1) and resource (row 2) densities under the HL scenario with heritable variation, constant variation, or no variation. The three trait variation scenarios when the patches are either connected (neighbouring patch is accessible) or isolated (neighbouring patch is not accessible) are compared over a range of maximum resource degradation rate D values. The varying (or evolving) trait is resource conversion efficiency ϵ. The no variation and constant variation lines are overlapping. Recall that resources do not have trait variation. The resource densities shown here are for the corresponding consumer trait variation scenarios. Note that the equilibrium densities are calculated for 21 equidistant points in each parameter range, but they are depicted by lines for better clarity. All other parameter values are at their default values from Tables 1 and 2.

We further explore the mutual effect that the patches have on each other, by running scenarios in which we artificially set consumer and resource density in the other patch to zero when determining foraging and mating for either patch (Figure 5). This setting represents isolated patches. We specifically focus on the effect of the resource removal rate D under all three variation scenarios. In the absence of HL (D=0), patch isolation has a positive effect on both consumer and resource density in the individual patches as well as on the landscape level (orange lines above turquoise lines at D=0 in Figure 5). This again is a consequence of overexploitation at lower consumer death rate ($a_{0}=0.1$ in Figure 5). In the isolated case, consumers still spend a fraction 1−p of effort searching for resources outside their patch which reduces the resource consumption pressure compared to the connected patches. As expected, the effect disappears at higher consumer death rates when the system is not in the overexploitation regime ($a_{0}=0.14$ in Figure S10).

As D, and thereby HL intensity increases to intermediate values, the pattern changes: Here, patch 1 consumers generally have higher equilibrium densities in the connected case compared to the isolated case. This happens likely because in patch 2, the consumers go extinct at a lower D value than the resources (Figure 5b,e), which provides cross‐foraging opportunities to patch 1 consumers in connected patches as long as there are some resources existing in patch 2 (D<0.4; Figure 5a,e). For patch 2, which directly experiences HL, foraging competition by patch 1 consumers in connected patches causes extinction of patch 2 consumers at even lower values of D as compared to patch isolation (Figure 5b, turquoise and orange lines; also see Figure S11b,e for corresponding results without the Allee effect where patch 2 consumers can survive higher D values). As a result of these observations in the individual patches, at the landscape scale, patch isolation gives higher consumer density at lower values of D, but as the value of D increases, patch connectedness gives a higher consumer density (barely visible in the heritable variation case), and finally the difference between the two disappears at even higher D values (Figure 5c, turquoise and orange lines).

With ϵ as the evolving trait, patch 2 consumers with heritable variation can withstand higher D values compared to the other two variation scenarios (Figure 5b, compare dotted lines with solid and dashed lines). Consumers with heritable variation also generally have higher equilibrium densities everywhere (Figure 5; also see Figure S9). Interestingly, with trait variation in $b_{1}$ (Figure S12), heritable variation leads to considerably lower equilibrium consumer densities at the landscape level, while still helping patch 2 consumers sustain higher D values (Figure S12b; dotted lines). Trait evolution also interacts with the impact of patch isolation. For example, at the landscape level, the difference between connected and isolated patches after the crossing of the orange and turquoise lines is not as prominent for the heritable variation scenario as for the no and constant variation scenarios (Figure 5c).

**FIGURE 6:**
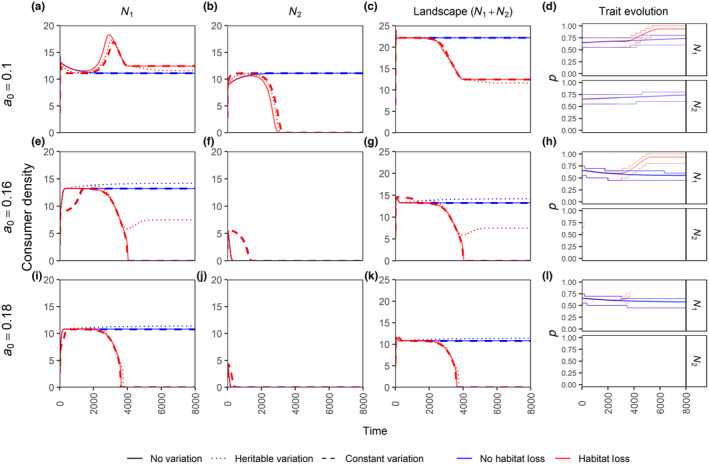
Time series plots of a scenario with coupled within‐patch foraging preference p and within‐patch mating preference β such that p=β. Patch 1 (panels a, e, i), patch 2 (panels b, f, j) and landscape‐level (panels c, g, k) consumer densities are shown for the three variation scenarios in the presence and absence of habitat loss. The last column (panels d, h, l) shows the trait evolution of p=β, where the central line denotes the mean trait value and the thinner outer lines denote the range which contains 90% of the density. Line transparency in the trait evolution plots denote consumer density. All other parameter values are default values from Tables 1 and 2, except for the per‐capita consumer death rate $a_{0}$ values, which are mentioned for each row.

Independent evolution of p, without corresponding evolution in β may be unlikely (although decoupling of foraging and mating could be possible when they occur at different places, like in systems with lek mating), because higher within‐patch foraging likely also implies higher within‐patch mating. We therefore explore the case where p and β are coupled, specifically p=β (Figure 6). Here, we have increased patch inter‐dependency by setting the initial within‐patch foraging (and mating) preference lower (p=β=0.65) than the default scenario. At lower death rates ($a_{0}=0.1$) and without HL, the consumers in the two patches can coexist, while at higher death rates ($a_{0}=0.16$ or 0.18) the consumers in the patch with higher initial density (here patch 1) outcompete the consumers in the other patch (Figure 6, blue lines; also see Figure S13 where patch 1 starts with lower initial density and gets out‐competed instead). Furthermore, without HL, the trait (p=β) evolves towards higher values when consumers in the two patches are coexisting (Figure 6d, blue lines) to avoid the reduced efficiency of cross‐foraging ($\epsilon_{c}$), and towards 0.5 when they are not coexisting (Figure 6h,l, blue lines) where consumers in a single patch can equally use resources in both the patches.

In the HL scenario, patch 2 consumers (and resources) go extinct (Figure 6 second column, red lines) and then the patch 1 consumers quickly evolve towards higher trait (p=β) values (Figure 6 fourth column) to maximize within patch resource use. This also shows that HL can impact the evolutionary trajectory of the evolving trait. Patch 1 consumers survive at the lowest death rate (Figure 6a, red lines), and when $a_{0}=0.18$ patch 1 always goes extinct (Figure 6i, red lines). At this high death rate patch 1 consumers rely on resources in patch 2 for survival; once these get degraded (HL case) the patch 1 consumers also suffer extinction. An interesting case occurs at intermediate death rates, where the extinction process is slow enough such that evolutionary rescue occurs in patch 1 for the heritable variation case only ($a_{0}=0.16$; Figure 6e red dotted line). Without heritable variation, these dynamics at the patch scale translate to the landscape scale such that, under the given parameter conditions, the consumer density in the whole landscape can go extinct after HL in a local patch (Figure 6g,k). Interestingly, this landscape scale extinction occurs even when HL is directly affecting the patch where the consumers are already extinct (Figure 6f,j). In this model, the Allee effect plays an important role in the landscape level extinction as we observe no such extinction in the absence of Allee effect (Figure S14).

## 4 DISCUSSION

In this study, we investigated the effects of habitat loss (HL) at the local (patch) and landscape scales in the presence or absence of trait variation (and evolution) using a two‐patch consumer‐resource system. Our results show that: (1) HL has detrimental effects for consumers in the directly affected patch and on the landscape as a whole. HL also often has negative effects on the adjacent patch, but it can also positively affect the consumer density in the adjacent patch when the resources are overexploited (Figure 2). (2) Patch connectedness can be detrimental to consumers in the patch experiencing HL and beneficial to the adjacent patch. Whether patch connectedness leads to higher consumer density on the landscape scale depends on the intensity of HL and the existence of overexploitation and/or evolution (Figure 5, Figure S10). (3) Trait evolution can have positive or negative effects on equilibrium population densities, depending on which trait is evolving and what scale is considered (Figures 4 and 5, Figure S12). (4) For those scenarios explored in our study, evolution allowed a patch directly affected by HL to sustain higher intensities of HL, irrespective of whether HL increases or decreases the consumer density in the adjacent patch or the landscape (Figure 5, Figure S12). (5) Lastly, we also show an example scenario where HL in a single patch can lead to consumer extinction at the landscape scale, which could be avoided (at low and intermediate consumer death rate) via evolutionary rescue (Figure 6). These findings highlight the importance of joint consideration of trait evolution and multiple spatial scales when predicting the effects of HL and patch isolation.

### 4.1 Multi‐scale context

The importance of choosing an appropriate spatial scale for ecological measurements has been extensively discussed in the literature (Holland & Yang, 2016; Levin, 1992; Wiens, 1989). Even HL and fragmentation have been found to have a scale‐dependent relationship with ecological response variables such as population status (sustained or declining) (Fuhlendorf et al., 2002), and habitat suitability (Treglia et al., 2018). Conservation and management is also benefited by simultaneous consideration of local and landscape scales (Bergman et al., 2012; Garden et al., 2010). However, the majority of these studies are related to differences in statistical measurements due to considering more or less area under the domain of analysis. Our results, on the other hand, not only show how effects of HL can differ at the patch and landscape scale, but also shed a light on how effects of HL can propagate from one patch to another, thus providing one possible explanation of why measurements can be sensitive to scale.

### 4.2 Cross‐patch effects

Habitat loss in patch 2 can either positively or negatively affect the consumer densities in the neighbouring patch (Figure 2). The negative effect can be attributed to a loss of cross‐foraging options. Potential positive effects occur because of reduced overexploitation through a reduction in the per‐capita consumption rate in patch 1 after HL (see Box 1 and SI Section S4 for details). Overexploitation happens when resources have logistic growth, and the consumers are highly efficient so that they end up pushing the resource density below its optimum growth rate (i.e. below half the carrying capacity $R^{*}<k/2$, see SI Section S4). In such cases, paradoxically, an apparent increase in consumer density may actually signal a deterioration of the system (Abrams, 2002). Overexploitation is a known property of consumer resource systems and has been shown in a model with two consumer species and two resource types before (Abrams, 2002), as well as in other modelling studies (Abrams, 2009, 2019; Prakash & de Roos, 2002). Unlike Abrams (2002), our study has mating, eco‐evolutionary dynamics, HL based on resource loss (not consumer parameters), and a spatial interpretation. Importantly, since many prey populations are below half of their carrying capacity (Abrams, 2019; Shurin et al., 2002), overexploitation is expected to occur frequently in nature assuming prevalence of logistic‐like density dependence in the populations (Abrams, 2019). Positive effects in the remaining patches have also been observed through other mechanisms such as edge effects (Ries et al., 2004) or in metapopulations through interaction of competitive ability and colonization‐extinction rates (Nee & May, 1992).

The reverse effect of the remaining patch on the patch experiencing HL (patch 2) is mostly detrimental, through cross‐patch competition. This effect pushes patch 2 consumers to extinction at even lower intensity of HL than without such competition (Figure 5). The possibility of negative effects of such cross‐patch competition has been researched using patch isolation experiments, for example, in a coral and coral associated fish system (Bonin et al., 2011), and a leaf litter and decomposing bacterial communities system (Spiesman et al., 2018). However, our results also show a possible rescue of patch 2 consumers in the HL scenario by the presence of a neighbouring patch when cross‐mating is common (β<0.5), while cross‐foraging is not (p=0.75, default value) (Figure 3e). Although unlikely, this scenario might occur under strong inbreeding avoidance, with individuals preferring to mate with individuals that are born elsewhere.

### 4.3 Patch isolation effects

The effects of patch isolation (or fragmentation) independent of habitat amount (or habitat loss) on ecological response variables such as species abundance and richness have been extensively discussed in the literature, where even positive effects of fragmentation have been found to be prevalent (Fahrig, 2003, 2013, 2017). We contribute to this discussion by showing that, under HL, patch isolation can have a positive effect on the local consumer density in the patch experiencing HL (patch 2) through reduced cross‐patch competition and a negative effect on density in the neighbouring patch (patch 1) through reduced cross‐foraging opportunities (Figure 5). On the landscape scale, which is generally where the fragmentation effects are measured (Fahrig, 2017), these opposing effects combined with overexploitation can lead to a positive effect of patch isolation on consumer densities at low HL intensity, a negative effect at intermediate, and no effect at higher HL intensity (Figure 5). However, this effect can become less prominent when consumers are evolving or completely disappear when the system is outside the overexploitation regime (Figure 5; Figure S10). Our study differs from previous studies in that HL only directly affects one of the patches, while in previous studies, the remaining habitat was typically redistributed, resulting in all patches losing some of their habitat (Bonin et al., 2011; Fahrig, 2017).

Current evidence for habitat fragmentation effects in conjunction with HL is inconsistent: theoretical studies predict negative effects at higher levels of HL (i.e. 20%–30% habitat remaining) (Bascompte & Solé, 1996; Fahrig, 1998), whereas empirical studies show both positive and negative (but majorly positive) effects at all levels of habitat availability (Fahrig, 2017; but see With, 2016). Some of the positive effects of fragmentation have been ascribed to release from competition or predation (Fahrig, 2017). Our study supports this reasoning for observations in patch 2, and further adds the release from resource overexploitation as another possible mechanism for positive effects of isolation in patch 1.

### 4.4 Effects of local HL on the landscape scale

Habitat loss is always detrimental to consumer density at the landscape scale, irrespective of its effects at the local scale (Figures 2 and 5). Thus, conserving as much habitat as possible should still be the priority. Furthermore, we also show the possibility of landscape‐level extinction when only one of the patches is experiencing resource loss, which can happen even when there are already no consumers in the patch directly affected by HL (Figure 6). This means that a naive management decision of allowing resources in a patch to be completely harvested because the species of interest (here consumers) does not produce any newborns in that patch can even cause landscape‐level extinction of the species. Hence, one should carefully consider the impacts of individual patch losses, as they can potentially lead to landscape level collapse. Furthermore, other mechanisms of environmental deterioration (sensu Abrams, 2002), which can independently affect consumer demographic parameters (such as consumption rate, death rate, and resource conversion efficiency), should also be taken into account. For example, at a lower per‐capita consumer death rate, the consumer population at the landscape level would still survive if one of the patch loses all the resources (Figure 6c), but if external habitat‐deteriorating factors increased the death rate of the consumers, then the same population would go extinct (Figure 6g,k). Our results also emphasize that the impacts of HL are exacerbated by an Allee effect since we observed landscape‐scale extinction in the presence of an Allee effect (Figure 6) but not in its absence (Figure S14) (also compare Figure 5b and Figure S11).

### 4.5 Effects of eco‐evolutionary dynamics

The eco‐evolutionary model (heritable trait variation) showed both quantitative (Figure 4) and qualitative (Figures 5 and 6) differences compared with the ecological model. Evolution of the consumption rate ($b_{0}$) and half saturation constant ($b_{1}$) even led to a decrease in the equilibrium consumer densities since the system was in an overexploitation regime. Such a reduction in densities is also referred to as ‘adaptive decline’ (Abrams, 2019). The constant variation scenario on the other hand yielded similar results as having no trait variation at all, except when the trait affects the consumer growth rates nonlinearly through Jensen's inequality (Jensen, 1906; Ruel & Ayres, 1999; Figure 4; Figure S12).

Heritable variation allowed the patch experiencing HL (patch 2) to survive higher D values as compared to other variation scenarios, through ‘evolutionary rescue’ (Bell & Gonzalez, 2011) (Figure 5b; also see Figure S12b, where the same phenomenon is observed even if heritable variation leads to lower equilibrium consumer densities). Furthermore, heritable variation allowed complete rescue of patch 1 when other variation scenarios went extinct (Figure 6, row 2). Evolutionary responses to HL and fragmentation have been studied theoretically, for example for evolution of dispersal distance (North et al., 2011) and coevolution of interacting species (Gawecka et al., 2022), as well as observed empirically. For example, selection of better colonization ability was observed in a large butterfly metapopulation in Finland (Fountain et al., 2016). However, there seems to be a lack of empirical evidence for such evolutionary responses specifically to HL to be rapid enough to save the population from extinction.

### 4.6 Assumptions, potential improvements, and a suggestion for an empirical test

Our model makes several assumptions. We consider the two‐patch consumer‐resource system as the simplest scenario to create a local (patch level) and landscape scale. The within‐patch foraging (p) and mating (β) parameters denote the traits that control the propensity to forage and mate in the patch of their birth. The patch boundaries do not have physical barriers so that the consumers can freely move in and out of the patch (similar to the ‘invisible’ boundary in Cantrell et al., 1998). Thus, our model could be relevant to freely moving consumer populations such as fish, birds, and insects with resources confined in their respective habitat patches.

Unlike previous two‐patch models with migration between patches (for example Gomulkiewicz et al., 1999; Gyllenberg et al., 1999; Jansen, 1995), our model does not assume direct transfer of population density between the patches. Instead, we have cross‐patch mating where half of the progeny is put in the adjacent patch. We also keep identical parameter values for the two patches, except that HL happens only in one of the patches, to avoid confounding the effects of HL with specific patch properties. However, traits in the two patches can evolve to different values in the evolutionary scenario. In principle, the patches could easily be made asymmetric based on other scenarios of interests such as source‐sink dynamics (Gomulkiewicz et al., 1999). Another point to be noted is that we have chosen the parameter ranges where the model shows stable equilibrium dynamics. One could indeed find regions with fluctuating dynamics in our model (for example, Figure S4) and extend the analysis in that direction (see, Jansen, 1995, 2001).

For simplicity, our study considers trait variation and evolution only in the consumer population, and the resource parameters are assumed to be constant. Given that the importance of studying the effects of environmental change under eco‐evolutionary dynamics is increasingly being emphasized (Åkesson et al., 2021; Faillace et al., 2021), the above assumption could be relaxed in future studies to consider consumer‐resource coevolution in the current model. In some of the trait evolution scenarios, traits evolve towards the minimum or maximum possible value, indicating that these evolutionary limits could be key parameters determining the long‐term response of populations to habitat loss. These parameters will be difficult to estimate for natural populations, but the extremes of the current distribution of individual traits or known physiological limits could be used as a first approximation. A further limitation of the current modelling approach is that only one trait can independently possess trait variation. If two uncoupled traits were to evolve, it could be done by considering a 2‐dimensional grid of bins, each representing a trait combination. Such a system could straightforwardly be extended to n traits. Although, in principle this is possible, it is outside the scope of this study and might be more easily achieved using an individual‐based model.

Our assumed system of inheritance, with offspring getting the mid‐parent trait value, also plays an important role in determining the evolutionary outcome. For example, in the scenario where p and β are coupled, one could expect that, under HL, patch 1 should evolve to p=β=1 (high within‐patch dependence) and patch 2 should evolve to p=β=0 (high cross‐patch dependence). However, we do not observe such divergent evolution in this case, or in any other scenario in our model, because the cross‐patch mating, mid‐parent value assumption, and non‐assortative (with respect to traits) mating together puts converging pressure on the evolving traits from the two patches. Dieckmann and Doebeli (1999) have shown such lack of evolutionary branching in an individual based model when there is sexual reproduction but without sufficient assortative mating.

Lastly, we propose a possibility to use the corals and coral‐associated fish system used by Bonin et al. (2011) to empirically validate some of our predictions, specifically regarding the effects of HL in one patch on the neighbouring patch, the effects of patch isolation interacting with HL intensity, and the possibility of landscape scale extinction due to HL in a local patch. Bonin et al. (2011) try to disentangle the effects of HL and fragmentation (patch isolation), where live corals are removed to mimic HL and the distance between experimental reefs is changed to manipulate patch isolation. In this setup, loss of live coral (resources) still leaves the coral beds, which can be accessed by the fish (consumers) as breeding sites, which satisfies one of our important assumptions. The amount of time spent in each patch for foraging and/or mating could be used as a proxy for their inherent preferences. Individual differences (variation) in these preferences and/or other consumer parameters (e.g. consumption rate) could further be used to understand the role of trait variation. Such empirical validation, either confirming or contradicting our theoretical predictions, would bring in the much needed dialogue between theory and experiment (Haller, 2014; Joshi, 2022; Otto & Rosales, 2020) as well as provide tangible predictions for future studies directly aimed at forming conservation policies.

## 5 CONCLUSION

Better assessment and prediction of the effects of HL would help in planning well‐informed conservation and management strategies. In our study, we demonstrate how HL and patch isolation can show opposite effects at the local patches and how they translate to the landscape scale, highlighting that any assessment of HL effects depends on the spatial scale at which the assessment is performed. Furthermore, Allee effect can exacerbate the impacts of HL. We also show how trait evolution can provide better resistance to HL in the affected patch, and under certain conditions even rescue the populations at the landscape scale. Our study should motivate future studies as well as conservation efforts to simultaneously consider multiple spatial scales and trait evolution while assessing and mitigating the effects of habitat loss.

## AUTHOR CONTRIBUTIONS

**Rishabh Bagawade:** Conceptualization (equal); formal analysis (lead); methodology (lead); writing – original draft (lead); writing – review and editing (equal). **Meike J. Wittmann:** Conceptualization (equal); formal analysis (supporting); funding acquisition (lead); methodology (supporting); supervision (equal); writing – review and editing (equal). **Koen J. van Benthem:** Conceptualization (equal); formal analysis (supporting); methodology (supporting); supervision (equal); writing – review and editing (equal).

## FUNDING INFORMATION

This study was partially funded by the German research foundation (DFG) as part of research consortium SFB‐TRR 212, project numbers 316099922 and 396782288.

## CONFLICT OF INTEREST STATEMENT

The authors declare no conflicts of interest.

## Supporting information


SI sections S1–S4, and figures S1–S14
R code to generate data and figures

## Data Availability

The R code for all the numerical simulations and figure generation can be found in the supplementary information section in the online version of this article.
